# Bio-Composite Nanogels Based on Chitosan and Hyaluronic Acid for the Treatment of Lung Infections

**DOI:** 10.3390/gels10110709

**Published:** 2024-11-01

**Authors:** Francesca Della Sala, Marco Barretta, Mario di Gennaro, Rubina Paradiso, Giorgia Borriello, Assunta Borzacchiello

**Affiliations:** 1 Institute of Polymers, Composites and Biomaterials, National Research Council (IPCB-CNR), Viale J.F. Kennedy 54, 80125 Naples, Italy; francesca.dellasala@cnr.it (F.D.S.); marco.barretta@unicampania.it (M.B.); mariodigennaro5@gmail.com (M.d.G.); 2 Department of Environmental, Biological and Pharmaceutical Sciences and Technologies (DiSTABiF), University of Campania “L. Vanvitelli”, 81100 Caserta, Italy; 3 Istituto Zooprofilattico Sperimentale del Mezzogiorno, Via Salute, 2, 80055 Portici, Italy; rubina.paradiso@izsmportici.it (R.P.); giorgia.borriello@izsmportici.it (G.B.)

**Keywords:** nanogels, chitosan, hyaluronic acid, antimicrobial activity

## Abstract

Pathogen infections constitute a serious problem in the field of lung diseases, especially in severe conditions such as chronic obstructive pulmonary disease (COPD) and acute respiratory distress syndrome (ARDS). Exacerbations of COPD and ARDS can be significantly influenced by bacterial infections from Pseudomonas aeruginosa and Staphylococcus aureus, which can hasten the decline of lung function. Moreover, the abuse of high-dose antibiotics used to treat obstinate infections is contributing to the growing issue of multidrug resistance (MDR) by microorganisms. Currently, new therapeutic strategies capable of surprising and fighting pathogens with new modalities are missing. In this framework, bio-composite nanogels (NGs) based on natural polymers with intrinsic antimicrobial properties such as chitosan (CS) and hyaluronic acid (HA) have been developed for the treatment of lung infections. The DLS and TEM results showed that NGs have a spherical shape with a size smaller than 100 nm, making it possible for them to potentially reach the lung site and evade the clearance of alveolar macrophages. FTIR spectra demonstrated that only electrostatic interactions, not chemical reactions, occur between NG precursors. Rheological analysis highlighted NGs’ injectability and mucoadhesive capacity. Moreover, an MTT assay on human lung fibroblast cells for biocompatibility evaluation showed good viability up to 48 h. Finally, an antimicrobial test on *P. aeruginosa* and *S. aureus* showed an increase in antimicrobial activity as the NG concentration increases, with a reduction in bacterial growth of around 60% at 375 μg/mL.

## 1. Introduction

In the area of respiratory illnesses, bacterial infections pose a substantial problem, particularly when treating acute respiratory distress syndrome (ARDS) and chronic obstructive pulmonary disease (COPD) [1]. Indeed, bacteria like *Haemophilus influenzae*, *Staphylococcus aureus*, *Pseudomonas aeruginosa*, and *Streptococcus pneumoniae* are frequently involved in bacterial infections in the setting of COPD and ARDS [2]. These infections exacerbate the already unstable respiratory condition of those afflicted with lung function impairment, raising the risk of morbidity and death. Adequate antimicrobial therapy is now lacking, particularly in light of the multidrug resistance (MDR) issue that bacteria are starting to exhibit [3]. This is mostly because of the overuse of antibiotics, their widespread application in agriculture, and the lack of new antibiotics. Moreover, high doses of antibiotics are related to unfavorable side effects, including allergic reactions, unintentional toxicity, alterations to the normal microbiota, and an increase in opportunistic infections [4].

Several studies have proven that biomaterial-based systems, like hydrogels, scaffolds, and membranes of polycationic materials, could function as either intrinsic medicines, if they have integrated antimicrobial properties, or as carriers for antimicrobial agents, offering potential new strategies to fight the effects of MDR [5]. Moreover, in the research into lung diseases, hydrogel-based biomaterials have gained much attention due to their physiological behavior mimicking the lung extracellular matrix (ECM) [6]. In this context, nano-sized hydrogels, which are three-dimensional networks of crosslinked hydrophilic polymers with a size smaller than 200 nm [7], have been explored with the aim of treating microbial infections. However, presently, most nanogels have been developed with the idea of acting exclusively as nanocarriers due to their ability to encapsulate antibiotics and other antimicrobial agents to prolong the targeted release effects. For example, enrofloxacin was encapsulated by oxidized hyaluronic acid-containing aldehyde groups and chitosan oligosaccharide-containing amino groups through Schiff’s base reaction to achieve release in the microenvironment of bacteria-infected wounds [8]. Nanogels for the co-delivery of nitric oxide and a novel antimicrobial peptide against bacterial biofilms have been developed [9]. Silver nanoparticles were embedded in a biocompatible nanogel comprising dextran and lysozyme [10]. Only a few works have studied the formulation of nanoparticles using natural polymers, such as chitosan as an antibacterial agent [11]. Lately, the development of nanogels with intrinsic antimicrobial moieties, such as quaternary ammonium compounds, guanidine, antimicrobial peptides, and their synthetic mimics, has been a topic of investigation but is still limited compared to the previously mentioned nanocarrier systems [12]. Thus, the nano-sized hydrogels manufactured to date do not exploit the intrinsic antimicrobial ability of natural polymers for biomedical application in the lung disease field, which could instead surprise the usual defenses of pathogens.

With this in mind, this study aimed to develop, for the first time, bio-composite nanogels (NGs) based on natural antimicrobial polymers such as chitosan (CS) and hyaluronic acid (HA), possessing intrinsic antimicrobial properties for the treatment of lung infections. Indeed, the nanometric size (<200 nm) of the bio-composites would allow them to reach the alveolar region of the lung more easily, evading the clearance of alveolar macrophages [13,14]. With this aim, the bio-composite CS/HA NGs were fabricated by self-assembly of the two natural polymers, avoiding the chemical modification of or alteration in the natural polymer structure. Indeed, antimicrobial natural polymers are materials capable of killing/inhibiting the growth of microbes due to their inherent capacity to display antimicrobial activity precisely thanks to their structure [15]. Among the natural biomaterials, CS is a linear polysaccharide containing a random distribution of β-(1–4)-linked D-glucosamine and N-acetyl-D-glucosamine repeating units originating from chitin, which is the second most abundant polysaccharide distributed in nature [16]. CS possesses intrinsic bactericidal properties due to the electrostatic interactions between its polycationic structure and the anionic groups found on the bacterial cell surface, leading to an alteration in cytoplasmic membrane permeability, followed by internal osmotic imbalances and, consequently, the inhibition of microorganism growth [17]. CS’s excellent qualities, including its biodegradability, biocompatibility, nontoxicity, non-antigenicity, and low production costs, have led to its extensive use in the medical, pharmaceutical, and food industries [18]. HA is a non-sulfate, non-immunogenic, biodegradable, and biocompatible anionic glycosaminoglycan (GAG) found ubiquitously in all of the body tissue extracellular and pericellular matrixes of vertebrates, including the lung ECM [19]. HA has inherent antibacterial activity since the units of HA are glucuronic acid (β(1–3) and N-acetylglucosamine (β(1–4) in alternating order, with an abundance of amide (CO–NH) and carboxyl (COOH) groups [20]. These groups offer a net negative charge, which causes the negatively charged bacterial cell wall to sterically repel, enhancing the bacteria’s ability to resist biocontaminants [21]. Furthermore, because HA plays a significant role in the ECM, the bacterial breakdown of it could have bacteriostatic consequences [22].

To obtain intrinsic antimicrobial NGs for the potential treatment of lung infection, we fabricated a bio-composite CS/HA NGs, generated by the interpenetration of natural polymer chains without chemical modification, using a simple ionic gelation method, by means of a syringe pump for a precise control of the flow and injection speed of the system. To this aim, the bio-composite CS/HA NGs were characterized for their size, superficial charge, and morphologies by dynamic light scattering (DLS) and transmission electronic microscopy (TEM), and their chemical composition was investigated by Fourier-transform infrared spectroscopy (FTIR). The stability of the CS/HA NGs was evaluated for 15 days at 4 °C and 37 °C. The mucoadhesive properties of the NGs and their injectability capabilities were characterized by rheological analysis. Eventually, their biological response on human lung fibroblast were investigated via a cell viability test, morphological analysis, and preliminary cell uptake investigation. The antimicrobial activity of the bio-composite NGs was tested on strains involved in lung infection diseases, such as *Staphylococcus aureus* and *Pseudomonas aeruginosa*. Overall, the data collected in this work indicated the potential use of such nano-sized bio-composite systems, which are intrinsically antimicrobial, for the treatment of lung infection.

## 2. Results and Discussion

### 2.1. Bio-Composite NG Characterization and Morphology

To obtain bio-composite NGs, CS and HA were chosen thanks to their ability to form nano-sized hydrogel with one-step ionic gelation. Indeed, the preparation of bio-composite NGs was based on physical crosslinking due to electrostatic interactions between the positive charge of CS and the negative charge of HA. In addition, TPP was used as a negatively charged crosslinker, and it was added to polymer mixtures to generate an interpenetrating polymer network. Indeed, the ionic gelation method is an attractive fabrication technique thanks to its mild processing needs, ease, and rapidity, as it requires a simple non-toxic aqueous environment. Moreover, the absence of a chemical reaction for NGs fabrication preserves the intrinsic properties of the precursors. Different NG formulations were investigated (Table 1). CS and TPP concentrations were maintained at constant values in every formulation, and their mass ratio was 2:1 (310 μg/mL and 155 μg/mL, respectively), while the HA concentrations were 7.0, 10, and 15 μg/mL, respectively (2.5, 3.3, and 5% *wt*/*wt* compared to CS amount).

**Figure 1:**
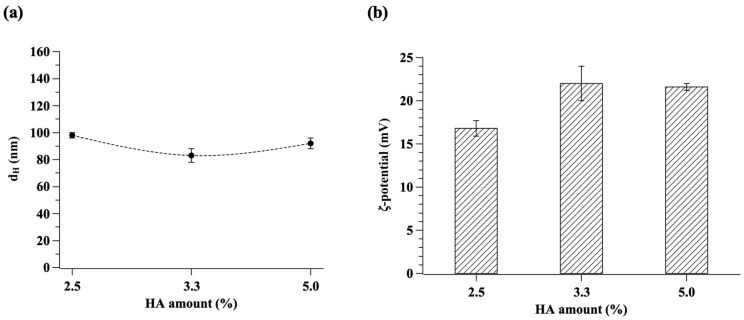
(**a**) Average hydrodynamic diameter (d_H_) and (**b**) *ζ*-potential of bio-composite CS-TPP/HA NGs at different amounts of HA. Data are the mean ± standard deviation of three independent experiments.

The average hydrodynamic diameter, PDI, and *ζ*-potential of bio-composite NGs were measured by DLS and are reported in Figure 1 and Table 2.

The data showed that the average hydrodynamic diameter is lower than 100 nm for all formulations investigated, hence deemed suitable for delivery in alveolar sacs [13,23]. In particular, the amount of HA varies the NG size as follows: 92 nm, 83 nm, and 98 nm for 2.5%, 3.3%, and 5.0% of HA, respectively. NG size does not change monotonically with the concentration of HA (Figure 1a and Table 2). Indeed, as HA is an anionic polymer with a long linear chain compared to TPP, it can present a high degree of entanglement and a less efficient packing of the polymer chains, resulting in less expulsion of water from the internal structure [24]. On the contrary, TPP is a small molecule with high anionic charge density, thus it interacts with the CS polymer chains, causing non-covalent crosslinking and improving the condensation [25,26]. For these reasons, because of HA acts as a polymeric crosslinker, the increase in its concentration leads to the two contrasting effect, conducting to the observed non-monotonic trend in the size.

PDI values (Table 2) vary from 0.267 to 0.34, indicating a relatively broad size distribution in accordance with the literature on CS-based NGs [8,27]. Indeed, the more the PDI values are closer to zero, the more the system can be considered homogeneous [28].

The *ζ*-potential values of NGs (Figure 1b and Table 2) are positive for all formulations due to the cationic charge contribution of CS, which is the major component of the NG structure compared to the anionic crosslinkers (TPP and HA). Moreover, the positive charge can be ascribed to the NG structure, assuming that the anionic crosslinkers are located inside the NGs structure, which expose mostly CS cation groups on their surface. Data show that *ζ*-potential goes from +16.8 ± 0.9 mV to +22 ± 2 mV, in agreement with the values reported in the literature for CS-only NGs [29], thus indicating that HA does not influence the surface charge of the systems.

**Figure 2:**
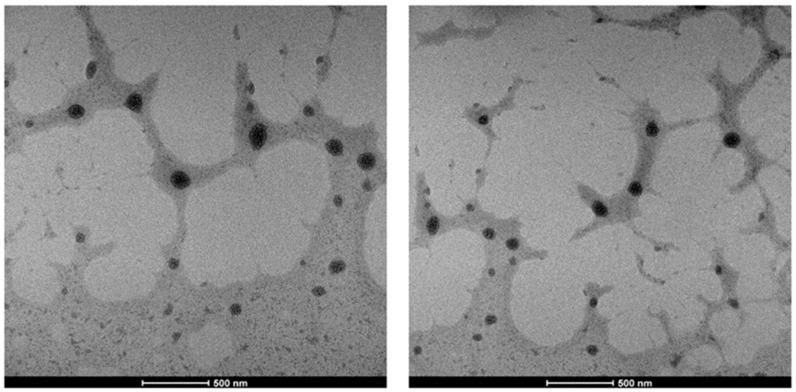
Representative TEM micrographs of CS-TPP/HA 3.3%.

The morphological characterization of the NGs were examined using TEM microscopy. Representative TEM micrographs of CS-TPP/HA 3.3% (Figure 2) showed that NGs, with discrete spherical shapes, were obtained with a regular morphology in the nano-sized range, with an average diameter of ~72 nm. The NGs appeared non-agglomerated and polydisperse. The average diameter of the NGs is smaller than the hydrodynamic diameter obtained by DLS measurements (Figure 1a) on NGs with the same polymer concentration (~83 nm). In accordance with the literature [30,31], this difference could be ascribed to the large amount of water contained in the NGs and the shrinkage of the NGs after water evaporation carried out for the sample TEM preparation.

### 2.2. FTIR Characterization

**Figure 3:**
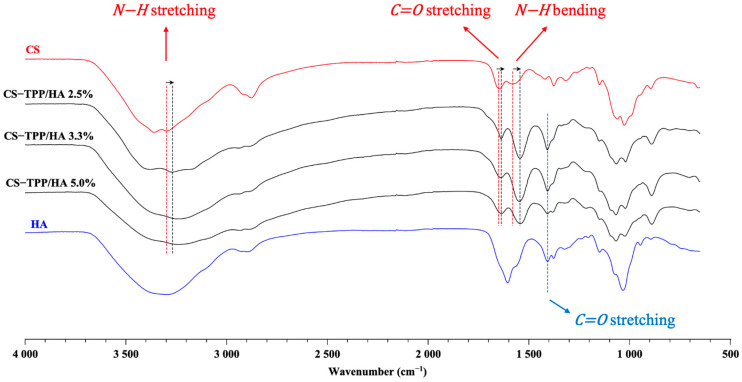
FTIR spectra of bio-composite NGs compared to HA and CS precursors spectra.

The ATR-FTIR spectra acquired from the different formulation of CS-TPP/HA NGs and the single components of the native HA and CS are shown in Figure 3. Figure S1 and Tables S1 and S2 report the precursor spectra and peak assignments for the CS and HA spectra, respectively.

The spectra of NGs are slightly different from the basic polymers, and they seem to be the sum of precursor spectra. The NG spectra showed peak shifts, but no new peaks appeared in any sample, showing that no new covalent bonds appeared. Indeed, NG formation is guaranteed by electrostatic interactions and not by chemical reactions. No notable differences were recorded between the spectra of the NGs at different HA contents, indicating that they have a very similar structure to each other. Due to the cross-linkage between CS and TPP, the peak at 3298 cm^−^^1^ shifted to 3270 cm^−^^1^ in NGs, as amino groups of CS played a role in this interaction [32]. Moreover, the shift of bands at 1650 cm^−^^1^ and 1580 cm^−^^1^ to 1637 cm^−^^1^ and 1544 cm^−^^1^, respectively, confirms bonding between amino groups of CS with phosphate groups of TPP [33]. The presence of the peak at 1407 cm^−^^1^, which is characteristic of carboxyl groups of HA confirmed the presence of HA in yjr NG structure. In this case, no shift was recorded, probably because the concentration of HA was very low; thus, the major crosslinking activity was insured by TPP.

### 2.3. Bio-Composite NG Stability over Time

**Figure 4:**
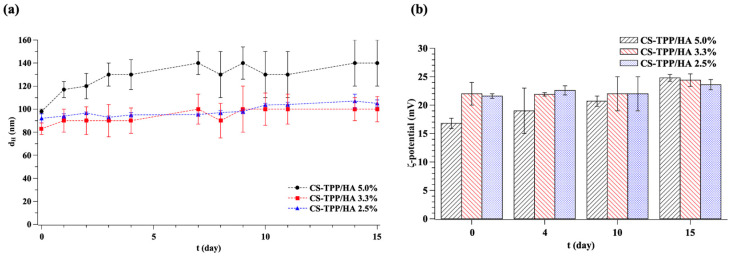
(**a**) Size and (**b**) *ζ*-potential over time of NGs stored at 4 °C for 15-day period after fabrication. Data are the mean ± standard deviation of three independent experiments.

**Figure 5:**
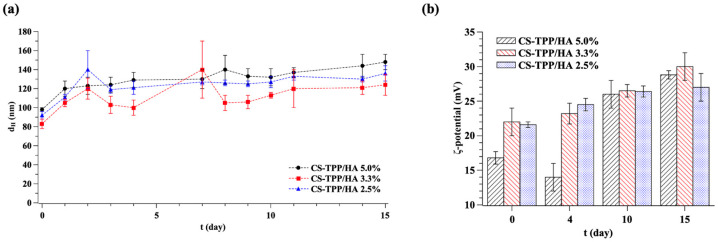
(**a**) Size and (**b**) *ζ*-potential over time of NGs stored at 37 °C for 15-day period after fabrication. Data are the mean ± standard deviation of three independent experiments.

In order to simulate the ordinary storage temperature and the physiological temperature of the body, the stability, in terms of size and *ζ*-potential of the NGs at three different formulations (2.5%, 3.3%, and 5.0% of HA), was assessed over a 15-day period after fabrication at 4 °C and 37 °C, respectively. Figure 4 shows stability data over 15 days at 4 °C, while Figure 5 shows stability data over 15 days at 37 °C.

The results showed that NGs stored at 4 °C remain quite stable during the investigated period. Indeed, CS-TPP/HA 2.5% and 3.3% NGs saw a diameter growth of ~20 nm, while NGs with 5.0% of HA saw a diameter growth of ~40 nm. It is likely that a higher concentration of HA generates less efficient condensation and an easier incorporation of water, which are determining factors of a change in size after 15 days.

All NGs showed a higher size growth when stored at 37 °C, with their sizes increasing to ~40 nm (for a HA content of 2.5% and 3.3%) and ~50 nm (for a HA content of 5.0%). As reported in the literature, CS hydrogels exhibit a temperature-responsive swelling behavior due to the temperature-dependent association/dissociation of hydrogen bonding between the amino groups within the CS chain [34]. The dissociation of hydrogen bonds between the amino groups of CS leads to more water diffusion in the hydrogel network and an increase in the swelling process. Even if this phenomenon is less significant in the presence of a crosslinking species [34], its effects are not nullified, justifying the higher diameter growth demonstrated when NGs are stored at 37 °C.

In accordance with size measurements, even the *ζ*-potential increases after 15 days at 4 °C and 37 °C. In particular, there is a small increase in NGs stored at 4 °C of about ~2 mV for 2.5% and 3.3% of HA and ~6 mV for 5.0% of HA, which is minor than in the case of 37 °C of about ~6 mV for 2.5%, ~8 mV for 3.3%, and ~12 mV for 5.0% of HA. The increase in *ζ*-potential may be explained either by the reorganization of the charged molecules within the NGs.

Instead, the PDI remains almost constant during the 15-day period at both 4 °C and 37 °C. Thus, based on these results, the temperature over time does not affect the homogeneity of samples, which increase their size without increasing their polydispersity.

### 2.4. Flow Curves and Mucoadhesivity of the Bio-Composite NGs

**Figure 6:**
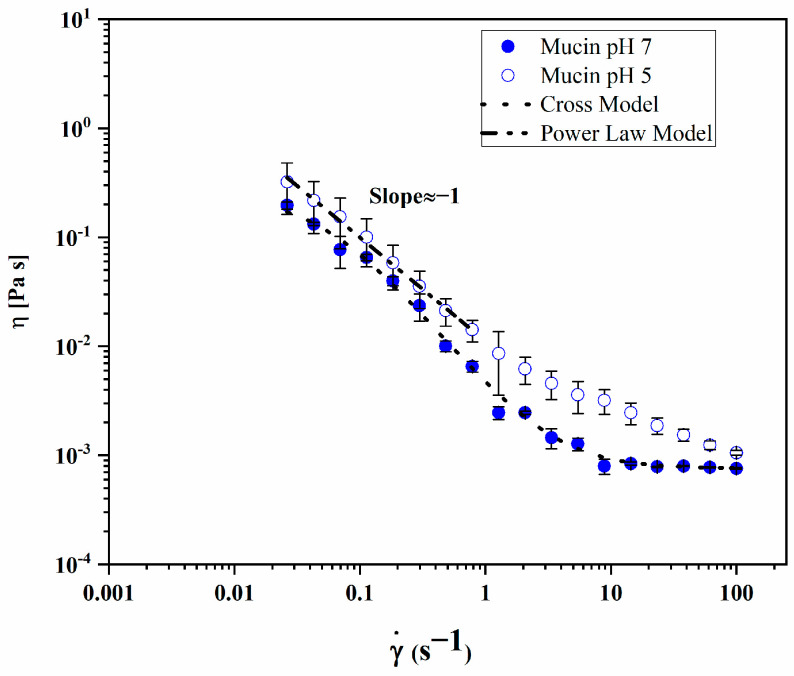
Flow curves of mucin at pH 7 (●) and at pH 5 (○).

The mucoadhesivity of CS-TPP/HA 2.5%, the formulation possessing the best performance in terms of stability, was investigated by means of rheological analysis. In order to analyze the mucoadhesivity of NGs, the flow curves of mucin, at pH 7 and 5, were first evaluated (Figure 6).

At both pH values, the solutions exhibited shear thinning but a different rheological behavior as follows: at pH 7, the mucin flow curve was interpolated with the Cross model (Equation (1)), that is commonly employed to describe the shear thinning behavior of fluids. In the bi-logarithmic scale, the slope of the shear thinning range of the curve is related to parameter m, and the higher it is, the more the fluid demonstrated shear thinning. As shear rate increases, mucin solution at pH 7 exhibits a zero shear viscosity η0 (0.25 Pa s), a decrease power law decrease (whose slope is related to the exponent m) and a infinite viscosity η∞ (7.6 10−4 Pa s), that according to the theory corresponds to the viscosity of water. At pH 5, in agreement with the data reported for other mucin solutions, the slope of the curve at a low shear rate suggests that the presence of a yield stress is necessary to deform the liquid [35]. The viscosity range at a low shear rate was interpolated with the Power Law model (Equation (2)). In the bi-logarithmic scale, n-1 is the slope of the straight line, which yielded ≈−1 in our data, in accordance with the data reported in the literature for mucin solutions at pH 4. The rheological parameters obtained by the fitting of mucin solutions are reported in Table 3.

**Figure 7:**
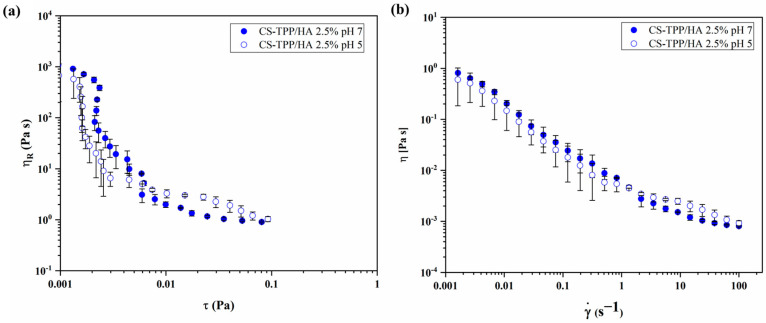
Flow curves of bio-composite CS-TPP/HA 2.5% NGs expressed as (**a**) reduced viscosity η_R_ as a function of shear stress τ and (**b**) viscosity as a function of shear rate, registered at pH 7 (●) and at pH 5 (○).

To analyze the rheological behavior of CS-TPP/HA 2.5%, Figure 7 shows reduced viscosity η_R_ as a function of shear stress τ (Figure 7a) and viscosity as a function of shear rate (Figure 7b), registered at pH 5 and 7. The relative viscosity η_R_, calculated with Equation (3), was used to express the contribution of CS-TPP/HA 2.5%. Compared to mucin, no significant changes were observed as an effect of pH change. The data reported in Figure 7a show how, from a qualitative point of view, the bio-composite NG suspensions exhibit a shear thinning behavior typical of interacting microgels [36]. Despite the low polymer concentration (0.4 mg/mL), the bio-composite NG showed a strong contribution of the viscosity of the suspension at low shear stress values, and a decrease in water viscosity over two decades of shear stress (from 0.001 to 0.1 Pa) was observed. The flow curves reported in Figure 7b exhibit a behavior similar to that of mucin, but neither traditional shear thinning models, such as Cross or Carreau, or models used for fitting hard sphere suspensions were suitable for the interpolation of our data. According to these results, it might be assumed that, at low values of the shear stress/rate, the interparticle interactions affect the viscosity of the suspension, and it strongly decreases as the shear stress/rate increases because of the low NG concentration. This aspect could be advantageous for pulmonary administration in the future application of NG-shaped materials. Indeed, the viscosity found in the range of 1 × 10^−3^ and 1 Pa s can be associated with practicability in the administration routes, such as via oro-tracheal or nasotracheal ventilation cannulas or via aerosolization through a cannula in the nose, to reach the pulmonary regions [37,38].

**Figure 8:**
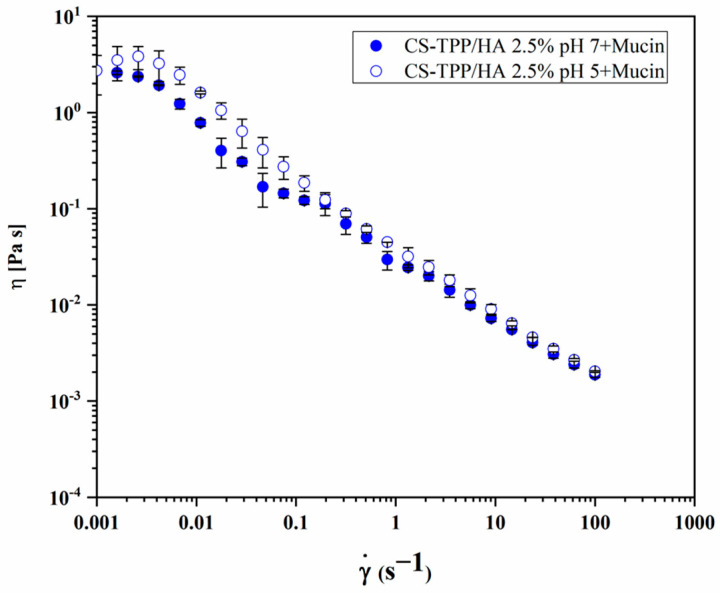
Flow curves of CS-TPP/HA 2.5% + mucin at pH 7 (●) and at pH 5 (○).

Then, the flow curves registered for CS-TPP/HA 2.5% + mucin at pH 7 and pH 5 are reported in Figure 8. The bio-composite NG–mucin mixtures exhibited higher viscosity compared to the single components throughout the analyzed shear rate range. Table 4 reports the values of viscosity measured at a shear rate of 0.01 s^−^^1^ at the two pH values and the viscosity component of the bioadhesion η_b_, calculated as reported in Equation (4), from the difference in the viscosity of the bio-composite NG + mucin mixture (η_mg_) compared to the sum of the viscosity of a single mucin (η_m_), and the NGs (η_g_). The results showed how η_b_ is higher than the viscosity of mucin and of the bio-composite NG, respectively. These data suggest that the interplay between mucin–bio-composite-NG contributes to the viscosity of the mixture more than the interaction of the single components themselves or with the solvent. The mucoadhesive properties of the polymers, such as chitosan, are affected by the chemical structure of polymers and the concentration of the reactive groups, like -NH_2_ and -COOH. These groups form non-covalent bonding with the mucin and can stick to the mucosal surface [39]. Thus, the favorable interaction with mucin could increase the performances of CS-TPP/HA 2.5% as a biomedical device against pulmonary infections.

### 2.5. Biological Response

**Figure 9:**
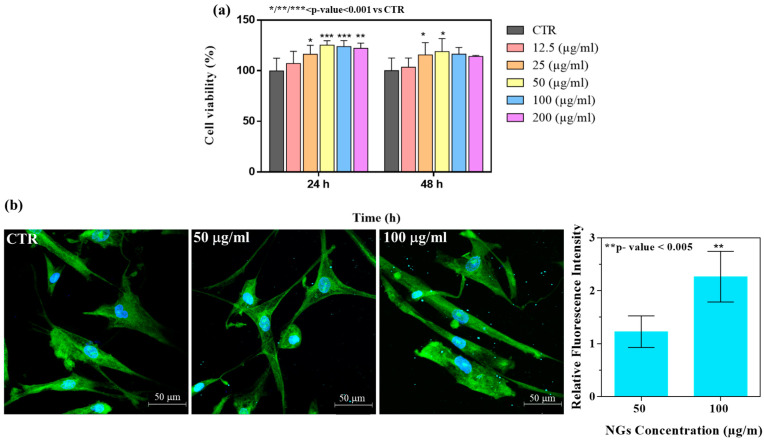
(**a**) Percentage of cell viability of HLF cells after 24 and 48 h of incubation with NGs at different concentrations. Cell viability was calculated with respect to the non-treated control cells (*^/^**^/^***p Value < 0.001 Vs CTR). (**b**) Representative confocal images of HLF cells exposed to 50 and 100 μg/mL of NGs after 24 h incubation. Maximum projection of Z-stack. Nuclei (DAPI) are shown in blue; actin filaments (phalloidin) in green; NGs in blue. Scale bar: 50 μm. NG internalization histograms after 24 h expressed as relative fluorescence intensity of the strained blue NGs.

A basic requirement for biomaterials intended for use in biomedical applications is cellular compatibility. In vitro biological characterization showed the biocompatibility of CS-TPP/HA 2.5%, which possessed the best performance in terms of stability over time. In particular, Figure 9a shows the cell viability percentage of HLF cells line after NG incubation at 24 and 48 h at different concentrations compared to the untreated control cells. The viability percentage increased to a value over 100% compared to the untreated control group for both 24 and 48 h of NG incubation, showing statistical significance for NG concentrations of 50 and 100 μg/mL. These results indicated that NGs not only possess the necessary safety but also that the CS and HA natural biopolymers can be easily metabolized by cells, leading to an overall beneficial effect on their viability. Indeed, the biocompatibility of CS is well known in the literature; in particular, it was reported that chitosan induced a significant effect on the growth promotion of normal skin fibroblasts [40,41]. In addition, HA as a physiological constituent of connective tissue was found to have a positive effect on cell viability, depending on its molecular weight and concentration [42]. The qualitative morphological analysis of cells was in agreement with the quantitate percentage measurement of viability. Indeed, representative confocal images of cells incubated with NGs at 50 and 100 μg/mL concentrations after 24 h and the control group without treatment (Figure 9b) show the typical healthy fibroblast shape of cells, elongated and with filopodia formation, as evidenced by the green staining of FITC phalloidin binding to cytoskeletal actin filaments [43,44]. Furthermore, the Alcian Blue staining of NGs allowed us to distinguish their internalization in cells. The qualitative cellular uptake of NGs after 24 h of incubation was shown as diffused blue dots in the cellular cytoskeleton. At the quantitative level, a preliminary analysis of internalization, measuring the intensity of the fluorescence signal of stained NGs displayed in the histograms, showed a higher internalization of the NG concentration at 100 μg/mL compared to 50 μg/mL.

### 2.6. Antimicrobial Activity

**Figure 10:**
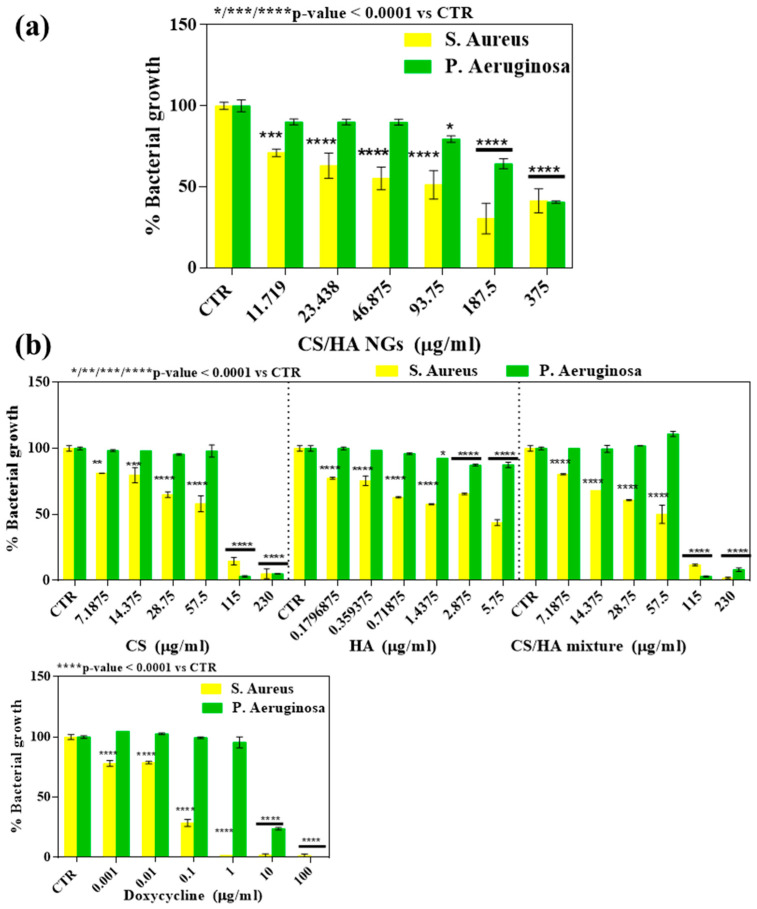
(**a**) Percentage of bacterial growth of Gram-positive *S. aureus* and Gram-negative *P. aeruginosa* after incubation of different concentrations of bio-composite NGs; (**b**) percentage of bacterial growth of control group, constituted from CS, HA, and CS/HA mixture solutions and Doxycycline, a broad-spectrum antibiotic. Percentage of bacterial growth was calculated compared to the non-treated bacteria CTR (*p*-value < 0.0001 vs. CTR).

The antimicrobial tests were performed on Gram-positive *S. aureus* and Gram-negative *P. aeruginosa*, both strains involved in lung disease, incubated with various concentrations (375,187.5, 93.75, 46.88, 23.44, and 11.72 µg/mL) of bio-composite NGs (Figure 10a). Moreover, in the control group, the antimicrobial effect of the precursor polymers was also demonstrated in the polymer solutions. In particular, solutions of CS (460 µg/mL), HA (11.5 µg/mL), and CS/HA mixture (460 µg/mL and 11.5 µg/mL, respectively) were tested. These concentrations were chosen to correspond with those of the individual polymers in the bio-composite NGs. Also, the antimicrobial activity of a broad-spectrum antibiotic named Doxycycline was tested (Figure 10b).

The results are expressed as the percentage of bacterial growth and demonstrate the efficacy of the bio-composite NGs in inhibiting the growth of the two strains. In particular, as shown in Figure 10a, a concentration-dependent effect of NGs can be noted in the reduction in bacterial growth %, which reaches about 40% at a concentration of 375 µg/mL. Furthermore, NGs were shown to have a better effect on the inhibition of bacterial growth on the *S. aureus* strain compared to *P. aeruginosa*. Indeed, already, at the lowest concentration of NGs used of 11,719 µg/mL, a decrease of 10% was found for *P. aruginosa*, while for *S. aureus* at the same concentration, bacterial growth was inhibited by 30%. The results obtained proved to be promising since bio-composite NGs are capable of killing both Gram-positive bacteria such as *S. aureus* and Gram-negative bacteria such as *P. aeruginosa*, due to their intrinsic antimicrobial capabilities. Antimicrobial tests were also performed on CS, HA, and CS/HA mixture solutions and on Doxycycline solutions, a broad-spectrum antibiotic, as the control group. The results shown in Figure 10b indicate that free Doxycycline is the most effective in inhibiting bacterial growth. In accordance with the literature, the polymers used alone are intrinsically antimicrobial. As can be seen, the CS-only solution has an inhibitory effect at concentrations of 115–230 µg/mL, as reported in the literature [45]. As is known, the HA solution instead showed a milder inhibitory effect than CS alone, especially on S. Aureus and less so on *P. aeruginosa*. The CS/HA mixture, in accordance with the ratio maintained in the gels between the two polymers, showed a trend more similar to CS than to HA, finding an additive effect of the two polymers particularly on *S. aureus*, especially at higher concentrations. Thus, in agreement with the literature, precursor polymer solutions were shown to be intrinsically antimicrobial. Comparing the solutions of the precursor polymers and the control antibiotic with the NG bio-composites, i in both cases, it was found that all compounds have a high inhibitory power on the Gram-positive *S. aureus* compared to the Gram-negative *P. aeruginosa* at all the concentration ranges tested. This could be attributed to the simpler structure of the membrane of the Gram-positive bacteria consisting of a single thick layer of peptidoglycan associated with negatively charged teichoic acid and lipoteichoic acid molecules. On the contrary, the *P. aeruginosa* strain seems to have a significant inhibition in its growth only when the tested compounds were at high concentrations. Interestingly, however, the NG bio-composites showed an inhibition that already appears significant at a lower concentration than in the tested solutions (93.75 µg/mL). This finding could be due to the presence of three barrier membranes in Gram-negative bacteria, including the hydrophobic outer membrane, the peptidoglycan layer, and the cell membrane, which hinder the infiltration of antimicrobial agents into the cells to interfere with DNA. Therefore, it is possible that only high doses of solutions can act significantly on this strain and that the nanometric shape may also allow internalization at lower doses. Possible mechanisms of action can be attributed to the structural characteristics of the bio-composite. Generally, it is well known that interactions between positively charged chitosan molecules and negatively charged microbial cell membranes lead to the leakage of protein and other intracellular constituents [15]. More specifically, the positive charge of bio-composite NGs, as demonstrated by the DLS analysis and the mucoadhesive properties of NGs able to interact with negatively charged mucin, indicated that such NGs present free amino groups that are able to bind to the negatively charged surface components of microbial cells, such as lipopolysaccharides present in Gram-negative bacteria and teichoic acids in Gram-positive bacteria [46]. The increased presence of these amino groups would cause a greater antimicrobial effect [47]. These results are in agreement with the dose-dependent trend observed both in the NG bio-composite groups and control groups of CS solutions only and the mixture. Moreover, antimicrobial activity is a function of pH, the pKa value of CS is approximately 6, and in the pH 7 condition, hydrophobic interaction and chelation effects can also contribute to biopolymer antimicrobial activity. Furthermore, the nano-sized form of bio-composite NGs with a size of 92 nm would allow the direct penetration of the cell wall of bacteria, with a size ranging from about 1 to 10 microns, and the combination with DNA, which directly affects synthesis of mRNA and DNA transcription [48]. Moreover, HA present in NGs could influence antimicrobial activity. Indeed, since HA is a major constituent of the ECM, bacteria are able to produce HA degrading enzymes. It can be assumed that this process could contribute to the bacteriostatic effects since the saturation of bacterial HA lyases, due to the presence of high MW HA in NGs, could lead to a decrease in bacterial growth [22]. In any case, while CS is only soluble in acetic media, and, therefore, not usable in biomedical applications, CS/HA NGs are easily dispersed in a distilled water medium for its good dispersity. The bio-composite NGs based on CS/HA in aqueous suspensions are consequently particularly promising since they were shown to possess an intrinsic antimicrobial effect on bacterial growth. Until now, research studies have described similar systems, i.e., CS- and HA-based nanosystems, that were used exclusively as carriers of antimicrobial agents, for example, in applications such as wound healing [8,49]. In this work, for the first time, the intrinsically antimicrobial role of a bio-composite NG based on CS/HA was investigated. The data collected here demonstrate that these devices can exploit the intrinsically antimicrobial capabilities of both natural polymers and that the nanometric shape preserved the structurally active portions capable of having a bacteriostatic action.

## 3. Conclusions

In this study, CS- and HA-based bio-composite NGs were fabricated by a single-step ionic gelation method, without chemical reactions between components, for the treatment of bacterial infections in lung disease. Physicochemical and morphological properties were performed on different NG formulations by means of DLS and TEM microscopy. The average hydrodynamic diameter of NGs tested by DLS was lower than 100 nm for all formulations investigated, with a positive surface charge indicating that, in the nanostructure, CS positive charge is arranged externally, while the HA negative charge is internal. TEM micrographs showed that spherical NGs were obtained with a regular morphology in the nano-sized range, resulting non-agglomerated and polydisperse NGs. The stability of the NGs up to 15 days were proven at 4 °C and 37 °C by DLS analysis. The FT-IR spectra of NGs were carried out to evaluate the nature of the interaction between CS and HA in the NG structure, and their spectra seem to be the sum of precursors. Indeed, small peaks shifts were recorded, and no additional peaks appeared, signaling the absence of chemical bonds between CS and HA and the presence of an electrostatic interaction only. Based on the dimensional and stability results, NGs with an amount of HA equal to 2.5% *wt*/*wt* appeared to be the most promising for the subsequent characterization. Rheological experiments demonstrated adequate injectability and mucoadhesive properties for lung therapy application. The biocompatibility of CS-TPP/HA 2.5% was demonstrated quantitatively by an MTT assay after incubation for 24 and 48 h with HLF cells and qualitatively by confocal microscopy. Finally, bio-composite NGs exhibited a good efficacy in bacterial growth inhibition for the *P. aeruginosa* and *S. aureus* bacterial strains. In particular, the bio-composite NGs showed a concentration-dependent antimicrobial activity for the concentrations at which an increase in HLF cell viability was found. Overall, these findings demonstrated the possibility of the employment of bio-composite NGs based on CS and HA for application in innovative antimicrobial therapy in the lung infection field. Furthermore, other studies can be carried out to better clarify how the CS/HA mass ratio affects the physicochemical properties of NGs in terms of their structural features. As a result, the design of bio-composites could be tuned to specific biomedical applications. Moreover, based on these preliminary results, the efficacy of the proposed system and its clinical applicability could be further confirmed by using in vivo models in future experiments. The focus is expected to be on the possibility of the employment of bio-composite NGs as alternative therapies for acute or chronic respiratory diseases due to bacterial infections and to overcome the MDR issue.

## 4. Materials and Methods

### 4.1. Materials

HA with an average molecular weight (M_w_) of 1510 kDa was provided by Altergon (Morra De Santis, Italy). High M_w_ chitosan (CS, M_w_ = 310–375 kDa), sodium tripolyphosphate (TPP), acetic acid (AcOH), bidistilled water and dimethyl sulfoxide (DMSO, 99.9% HPLC grade), and fibroblast growth medium were purchased from Sigma Aldrich (Burlington, Massachusetts, USA). Phosphate-buffered saline (PBS) tablets without calcium and magnesium were obtained from MP Biomedicals Inc. (Eschwege, Germany). Penicillin and streptomycin (P/S, 10,000 U/mL) from Invitrogen and Life Technologies (Carlbad, CA, USA) were employed. Trypsin was purchased from HiMedia (Mumbai, India). Fetal Bovine Serum (FBS) from Lonza (Base, Switzerland). Human lung fibroblast (HLF) cell line was purchased from ATCC (Manassas, VA, USA).

### 4.2. Bio-Composite CS-TPP/HA NG Fabrication

CS-TPP/HA NGs were fabricated with the ionic gelation method using a syringe pump for the precise control of the flow and injection speed of the system [50]. Briefly, 2.365 mL of HA was mixed with 365 μL of TPP solution. Three different formulations were prepared with different HA concentrations (15.4, 20.5, and 30.7 μg/mL), whereas the TPP concentration was constant in every formulation (2 mg/mL). To obtain NGs, the full amount of the HA/TPP mixture was added drop by drop using a syringe pump (0.5 mL/min) with a gauge of 23 G 1 ¼”, diameter 0.60 × 30 mm, to 2 mL of CS solution (0.727 mg/mL in AcOH 2% *v*/*v*) under strong magnetic stirring. Through this method, ionic gelation occurred because of the electrostatic interaction between the negatively charged HA/TPP mixture and the positively charged CS. Finally, the obtained NG suspension was dialyzed against water using a dialysis membrane tube of 12–14 kDa for 24 h to eliminate the AcOH residues. The final HA concentration (%*w*/*w* respect to CS amount) varied from 2.5%, 3.3%, to 5%, whereas the CS/TPP mass ratio was 2:1 for every formulation.

### 4.3. Dynamic Light Scattering (DLS)

Intensity-average hydrodynamic radius and ζ-potential of NGs were detected by means of dynamic light scattering (DLS) measurements with a Zetasizer Nano (Malvern Instrument, Malvern, UK). To investigate agglomeration dynamics, swelling capacity, and stability over time, NGs were stored in bidistilled water at 4 °C and 37 °C, considering the ordinary storage condition and the physiological temperature. Measurements were acquired in triplicate for 15 days.

### 4.4. Transmission Electron Microscopy (TEM)

NG morphology was investigated by means of TEM (FEI Tecnai G12 Spirit Twin) with emission source LaB6 (120 kV, spot size 1) using 400 mesh carbon-coated copper grids at room temperature (RT). The carbon-coated copper grid was immersed in ultra-diluted NG suspensions, and, after the drying phase, the grid was placed on a rod holder for TEM characterization. Three grids per NP suspension were prepared, and a minimum of four micrographs per grid were acquired.

### 4.5. Fourier Transform Infrared Spectroscopy (FTIR)

Fourier-transform infrared spectroscopy (FTIR, ATR Perkin Elmer) was performed on HA and CS powders and CS-TPP/HA NGs (2.5%, 3.3% and 5.0% of HA) to assess the functional groups of the polymers and to evaluate their interaction after NG formation. The acquisition range was from 4000 to 650 cm^−1^ with a resolution of 1 cm^−1^ for each sample. A total of 64 scans were performed for each sample to obtain an adequate signal-to-noise ratio.

### 4.6. Rheological Characterizations

The mucoadhesivity of CS-TPP/HA 2.5% suspension was investigated by means of rheological analysis using a rotational rheometer (RheoStress 6000, HAAKE Rheometer, Waltham, MA, USA) equipped with cone–plate geometry [51]. Steady shear analysis was performed by measuring viscosity η as a function of shear rate $\dot{\gamma }$ in the range 10^−3^–100 s^−1^ at 25 °C. For each test, the shear stress τ at each shear rate value was measured. The flow curves of CS-TPP/HA 2.5%, mucin, and CS-TPP/HA 2.5% + mucin at the compositions reported in Table 5 were registered at 37 °C and pH 5 and 7, respectively.

The Cross model (Equation (1)) and Power Law model (Equation (2)) were used to interpolate mucin flow curve at pH 7 and 5, respectively.

(1) $$\eta =\eta_{\infty }+\frac{\eta_{0}-\eta_{\infty }}{1+{\left(\lambda \dot{\gamma }\right)}^{m}}$$

(2) $$\eta =k{\dot{\gamma }}^{n-1}$$

where η_0_ and η_∞_ are the viscosity of the liquid at the low shear rate of a Newtonian plateau and at the high shear rate of a Newtonian plateau; λ is the transition time and a time constant of the equation; m is the exponent term that determines the flow behavior of the system; k is the flow consistency index; and n is the flow behavior index.

The relative viscosity η_R_, calculated by means of Equation (3), was used to express the contribution of CS-TPP/HA 2.5% to solvent viscosity as follows:

(3) $$\eta_{R}=\frac{\eta }{\eta_{S}}$$

where η_S_ is the viscosity of the solvent.

Finally, the viscosity component to the bioadhesion η_b_ was calculated as reported in Equation (4).

(4) $$\eta_{b}=\eta_{mg}-(\eta_{m}+\eta_{g})$$

where η_m_, η_g_, and η_mg_ are the viscosities of mucin, of the bio-composite NG, and of the mucin–NG mixture, respectively. The flow curves were registered at pH 5 and 7 in order to evaluate the effect of the protonation of chitosan (pKa = 6.5) on the mucoadhesion of the nanogel.

### 4.7. Biological Response

#### 4.7.1. Cell Culture

Human lung fibroblast (HLF) cells were grown in T-75 cell culture flask (Corning Falcon, Arizona, USA) in the fibroblast growth medium implemented with 1 ng/mL insulin at 37 °C and 5% CO_2_. When confluent growth was reached, the cells were washed twice with PBS and detached with 0.25% trypsin-EDTA solution. The resulting cell suspensions were centrifuged (5 min, 1200 rpm, BRK55/10 Centrifuge by Centurion Scientific Ltd., (Chichester, UK), the supernatant was separated, and the cells were re-suspended in a fresh culture medium. Viable cells were counted using the TC20 automated Cell Counted (Bio-Rad, Hercules, California, USA).

#### 4.7.2. Cell Viability

In order to evaluate the viability of cells once incubated with NGs, the quantitative colorimetric MTT assay was carried out. HLF cells were seeded onto 96-well culture plates at a density of 1.2 × 10^4^ cells per well and left to adhere in 200 μL of the culture medium overnight. Afterwards, the cells were incubated with NG suspension in the fresh culture medium at different concentrations (12.5, 25, 50, 100, and 200 mg/mL). After 24 and 48 h, the metabolic activity was measured adding 3-(4,5-dimethylthiazol-2-yl)-2,5-diphenyl tetrazolium bromide (MTT, 0.25 mg/mL in culture medium). The tetrazolium salt was reduced into formazan (insoluble) by metabolically active cells using mitochondrial succinate dehydrogenase enzymes. The MTT solution was carefully removed from each well, and the resulting dark blue formazan crystals were solubilized in DMSO and quantified spectrophotometrically (Multilabel Counter, 1420 Victor, Perkin Elmer, Hopkinton, MA, USA) at 570 nm. The experiments were performed in triplicate, and the metabolic activity results were shown as a percentage of cell viability, estimated with Equation (5), which outlines the plates at a density of 1.2 × 10^4^ cells per well and left adhering in 200 μL of culture medium as follows:

(5) $$Cell\;viability\left(\%\right)=\left(\frac{A_{s}}{A_{c}}\right)\times 100$$

where A_s_ points to the absorbance of cells samples treated, and A_c_ is the absorbance of normal untreated cells.

#### 4.7.3. Cell Morphology and NG Uptake

The cell morphology of HLF cells was evaluated by seeding them at a density of 1 × 10^4^ cells/mL on Chamber Slide System (Thermo Scientific™ Nunc™ Lab-Tek™, Waltham, Massachusetts, USA). NGs were formulated by previously staining the HA chains with Alcian Blue. Briefly, glacial acetic acid (30% *v*/*v*) and ethanol (70% *v*/*v*) at pH ~2 were added to Alcian Blue (1% *w*/*v*), and this solution was added (1:100) to the HA solution and stirred for 30–40 min at RT. Then, NGs were obtained with same protocol described above. The NGs at concentrations of 50 and 100 µg/mL were incubated with cells for 24 h. After incubation, cells were rinsed three times with PBS to remove non-internalized NGs and fixed with 10% formaldehyde for 1 h at 4 °C. Cells were permeabilized with Triton X-100 0.1% in PBS for 3–5 min, and the actin filaments were stained with FITC phalloidin/PBS for 30 min at RT. Finally, after two washes with PBS to remove the unbound phalloidin, cell nuclei were stained with 4′,6-diamidino-2-phenylindole DAPI, (Sigma Aldrich, St. Louis, Missouri, United States). The cells were observed using a confocal microscope system (Leica TCS SP8) with a 63× oil immersion objective. Excitation lasers were set to work at 480 nm for Alcian Blue, while emission detection ranges were set in the interval 464–486 nm for Alcian Blue. Images were acquired with a resolution of 1024 × 1024 pixels by z-stacking, and the thickness of each z-slice was set to be equal to 0.1 μm. To evaluate the NG uptake, fluorescent images were imported into LAS X software version 1.4.6 28433 (Wetzlar, Germany) for post-processing analysis and uptake quantification. Single cells were delineated by identifying a ROI per single cell to eliminate fluorescent NGs outside the cell. The semiquantitative mean fluorescent intensity for the channel of Alcian Blue within the cell boundaries was calculated by LAS X software version 1.4.6 28433 (Wetzlar, Germany) [28].

### 4.8. Antimicrobial Test

Overnight cultures of Staphylococcus aureus or Pseudomonas aeruginosa grown in Brain Heart Infusion Broth (BHI, Oxoid, ThermoFisher Scientific, Waltham, Massachusetts, USA), with an incubation temperature of 37 °C were used to prepare bacterial cell suspensions for antibacterial activity tests. Bacterial cells were washed twice with saline solution and adjusted to a turbidity level equal to 0.5 McFarland standard (BioLife, Milano, Italy). The bacterial growth measurement was performed in 96-well plates. Each well contained 20 µL of bacteria cell suspension and various concentrations (375,187.5, 93.75, 46.88, 23.44, and 11.72 µg/mL) of bio-composite NGs suspended in BHI medium. Moreover, to assess the antimicrobial effect of the precursor polymers, the same measurements were carried out on polymer solutions. In particular, solutions of CS (460 µg/mL), HA (11.5 µg/mL), and the mixture of CS and HA (460 µg/mL and 11.5 µg/mL, respectively) were tested. These concentrations were chosen to coincide with those of individual polymers in bio-composite NGs. The antimicrobial activity of bio-composite NGs were compared with the effect of a broad-spectrum antibiotic named Doxycycline (100 µg/mL). The analysis was performed in triplicate. The treated bacterial samples were then incubated at 35 °C for 24 h. The optical densities (ODs) of the samples were measured at wavelength of 600 nm after incubation using Multiskan™ FC Microplate Photometer (ThermoFisher Scientific, Waltham, Massachusetts, USA). The inhibitory effect of treatment on bacterial growth was evaluated based on a decrease in the OD600 values compared to those for the untreated control samples. The percentage of bacterial growth was calculated by dividing the OD of each sample for the OD of the untreated control group.

### 4.9. Statistical Analysis

The results were expressed as the mean ± standard deviation (SD). Data analysis was performed using Graphpad^®^ Prism 6 software. The repeated results were compared with the ordinary 2-way analysis of variance (ANOVA). For the cell viability test, *p*-value < 0.001 was considered significant, and for the t test, *p*-value < 0.005. For the antibacterial activity test, Tukey’s multiple comparisons test was carried out, with *p*-value < 0.0001 considered significant.

## Figures and Tables

**Table 1:** Components concentration (μg/mL) in the NGs for each formulation.

Sample	CS (μg/mL)	TPP (μg/mL)	HA (μg/mL)
CS-TPP/HA 2.5%	310	155	7.0
CS-TPP/HA 3.3%	310	155	10
CS-TPP/HA 5.0%	310	155	15

**Table 2:** Average hydrodynamic diameter (d_H_) and (b) *ζ*-potential CS-TPP/HA at different amount of HA. Data are the mean ± standard deviation of three independent experiments.

Sample	d_H_ (nm)	PDI	ζ-Potential (mV)
CS-TPP/HA 2.5%	92 ± 4	0.34 ± 0.04	+21.6 ± 0.4
CS-TPP/HA 3.3%	83 ± 5	0.33 ± 0.05	+22.0 ± 2.0
CS-TPP/HA 5.0%	98 ± 2	0.267 ± 0.004	+16.8 ± 0.9

**Table 3:** Rheological parameters obtained by fitting the mucin flow curves.

Sample	Cross Model			
Mucin pH 7	η_0_ (Pa s)	η_∞_ (Pa s)	λ (s)	m (−)
	0.25 ± 0.04	(7.6 ± 0.1) × 10^−4^	20 ± 5	1.37 ± 0.06
	**Power Law model**			
Mucin pH 5	λ (Pa s^n^)		n (−)	
	0.0116 ± 0.0006		0.1 ± 0.02	

**Table 4:** Viscosity (η_g_, η_m_, η_mg_, and η_b_) at a shear rate of 0.01 s^−1^.

	η_g_ (Pa s)	η_m_ (Pa s)	η_mg_ (Pa s)	η_b_ (Pa s)
pH 7	0.203 ± 0.013	0.25 ± 0.09	0.78 ± 0.06	0.33 ± 0.09
pH 5	0.15 ± 0.09	0.5 ± 0.3	1.61± 0.05	1.61± 0.05

**Table 5:** Concentration of samples analyzed in mucoadhesivity tests.

Sample	CS-TPP/HA 2.5% (mg/mL)	Mucin (mg/mL)
CS-TPP/HA 2.5%	0.4	0
Mucin	0	1
CS-TPP/HA 2.5% + Mucin	0.4	1

## Data Availability

The data presented in this study are openly available in the article.

## Supplementary Materials

The following supporting information can be downloaded at: https://www.mdpi.com/article/10.3390/gels10110709/s1, Figure S1: FTIR spectra of (a) CS and (b) HA powders, Table S1: Peak assignment for CS FTIR spectrum, Table S2: Peak assignment for HA FTIR spectrum, Table S3: Size and PDI over time of NGs at different amounts of HA (2.5%, 3.3%, 5.0%) and stored at 4 °C, Table S4: ζ-potential over time of NGs at different amounts of HA (2.5%, 3.3%, 5.0%) and stored at 4 °C, Table S5: Size and PDI over time of NGs at different amounts of HA (2.5%, 3.3%, 5.0%) and stored at 37 °C, Table S6: ζ-potential over time of NGs at different amounts of HA (2.5%, 3.3%, 5.0%) and stored at 37 °C.
