# Supplementary material for: Bio-Composite Nanogels Based on Chitosan and Hyaluronic Acid for the Treatment of Lung Infections
Source: Gels. 2024 Nov 1;10(11):709. doi: 10.3390/gels10110709 (PMC11594208; doi:10.3390/gels10110709)
Supplement: Supplementary file 1 [file gels-10-00709-s001.zip › gels-3255050-supplementary.pdf]

Supporting information

Bio-composite Nanogels Based on Chitosan and Hyaluronic Acid for the Treatment of Lung Infections

Francesca Della Sala[1†], Marco Barretta[1,2†], Mario di Gennaro[1], Rubina Paradiso[3], Giorgia Borriello[3] Assunta Borzacchiello[1*]

[1]Institute of Polymers, Composites and Biomaterials, National Research Council (IPCB-CNR), Viale J.F. Kennedy 54, 80125, Naples, Italy
[2]Department of Environmental, Biological and Pharmaceutical Sciences and Technologies (DiSTABiF), University of Campania "L. Vanvitelli", 81100 Caserta, Italy
[3] Istituto Zooprofilattico Sperimentale del Mezzogiorno, Via Salute, 2, 80055, Portici, Italy

* correspondence to: Dr. Assunta Borzacchiello: bassunta@unina.it, assunta.borzacchiello@cnr.it

[†]These authors contributed equally to this work.

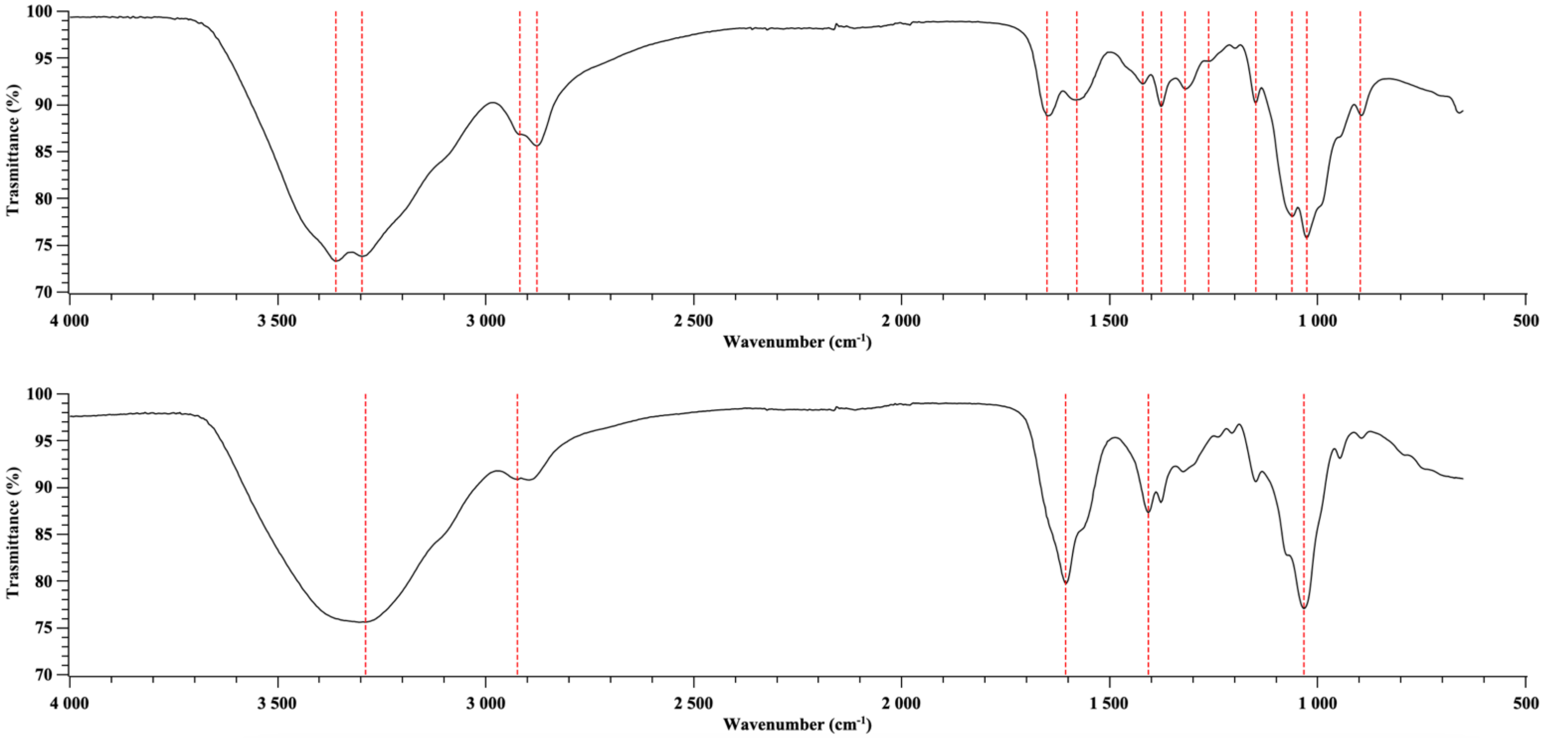

Figure S1: FTIR spectra of (a) CS and (b) HA powders. Characteristic peaks are highlighted. The acquisition range was from 4000 cm^{-1} to 650 cm^{-1} with a resolution of 1 cm^{-1} and 64 scans for each sample.

Table S1: Peak assignment for CS FTIR spectrum.

**Peak center (cm^{-1})**	**Molecular vibration**
3361	$O-H$ and $N-H$ stretching
3298	$O-H$ and $N-H$ stretching
2919	$C-H$ symmetric stretching
2877	$C-H$ asymmetric stretching
1650	$C=O$ stretching of amide I
1580	$N-H$ bending of primary amine
1421	$-CH_2$ bending
1376	$-CH_3$ symmetrical deformations
1319	$C-N$ bending of amine III
1261	$O-H$ bending
1149	$C-O-C$ asymmetric stretching
1061	$C-O$ stretching
1027	$C-O$ stretching
897	$C-H$ bending out of the plane

Table S2: Peak assignment for HA FTIR spectrum.

Peak center (cm^{-1})	Molecular vibration
3289	$O-H$ and $N-H$ stretching
2924	$C-H$ symmetric stretching
1606	$C=O$ stretching of amide I
1407	$C=O$ stretching of carboxyl groups
1033	$C-O$ stretching oh alcohol

Table S3: Size and PDI over time of NGs at different amount of HA (2.5%, 3.3%, 5.0%) and stored at 4 °C. Data are the mean ± standard deviation of three independent experiments.

	d_H (nm)			PDI		
**t (day)**	**2.5%**	**3.3%**	**5.0%**	**2.5%**	**3.3%**	**5.0%**
**0**	92 ± 4	83 ± 5	98 ± 2	0.34 ± 0.04	0.33 ± 0.05	0.267 ± 0.004
**1**	94 ± 2	90 ± 10	117 ± 7	0.31 ± 0.04	0.30 ± 0.03	0.28 ± 0.03
**2**	96.6 ± 0.9	90 ± 12	120 ± 11	0.33 ± 0.03	0.31 ± 0.03	0.30 ± 0.06
**3**	92.9 ± 1.2	90 ± 14	130 ± 10	0.35 ± 0.02	0.31 ± 0.03	0.31 ± 0.05
**4**	95 ± 2	90 ± 11	130 ± 13	0.32 ± 0.05	0.32 ± 0.05	0.32 ± 0.05
**7**	95.3 ± 1.2	100 ± 13	140 ± 10	0.35 ± 0.03	0.34 ± 0.09	0.33 ± 0.06
**8**	97 ± 2	90 ± 15	130 ± 20	0.34 ± 0.04	0.32 ± 0.02	0.30 ± 0.05
**9**	98 ± 2	100 ± 20	140 ± 14	0.34 ± 0.04	0.31 ± 0.06	0.30 ± 0.05
**10**	103.6 ± 1.5	100 ± 14	130 ± 20	0.35 ± 0.04	0.32 ± 0.03	0.31 ± 0.07
**11**	104 ± 2	100 ± 13	130 ± 20	0.35 ± 0.06	0.36 ± 0.05	028 ± 0.03
**14**	107 ± 6	100 ± 10	140 ± 20	0.34 ± 0.05	0.34 ± 0.05	0.30 ± 0.04
**15**	105 ± 3	100 ± 11	140 ± 20	0.36 ± 0.04	0.35 ± 0.06	0.31 ± 0.04

Table S4: ζ-potential over time of NGs at different amount of HA (2.5%, 3.3%, 5.0%) and stored at 4 °C. Data are the mean ± standard deviation of three independent experiments.

	**ζ-potential (mV)**		
**t (day)**	**2.5%**	**3.3%**	**5.0%**
**0**	21.6 ± 0.4	22 ± 2	16.8 ± 0.9
**1**	22.8 ± 0.6	21.2 ± 0.4	19.2 ± 0.8
**2**	22 ± 1	21.2 ± 0.6	20.0 ± 0.3
**3**	22 ± 1	22 ± 1	19.9 ± 0.5
**4**	22.6 ± 0.8	20.9 ± 1.3	19 ± 4
**7**	23.1 ± 0.6	21.6 ± 1.1	22 ± 2
**8**	23 ± 2	23.8 ± 1.2	22 ± 2
**9**	23.0 ± 0.3	23 ± 2	22.1 ± 0.6
**10**	23 ± 3	22 ± 3	20.7 ± 0.9
**11**	21 ± 3	22 ± 3	22.3 ±1.4
**14**	22 ± 2	23 ± 2	24 ± 2
**15**	23.6 ± 0.9	24.4 ± 1.1	24.8 ± 0.6

Table S5: Size and PDI over time of NGs at different amount of HA (2.5%, 3.3%, 5.0%) and stored at 37 °C. Data are the mean ± standard deviation of three independent experiments.

	**d_H (nm)**			**PDI**		
**t (day)**	**2.5%**	**3.3%**	**5.0%**	**2.5%**	**3.3%**	**5.0%**
**0**	92 ± 4	83 ± 5	98 ± 2	0.34 ± 0.04	0.33 ± 0.05	0.267 ± 0.004
**1**	111 ± 3	105 ± 4	120 ± 8	0.30 ± 0.01	0.32 ± 0.04	0.28 ± 0.03
**2**	140 ± 20	120 ± 11	123 ± 9	0.33 ± 0.03	0.29 ± 0.03	0.30 ± 0.06
**3**	119 ± 4	103 ± 9	124 ± 8	0.33 ± 0.01	0.33 ± 0.02	0.31 ± 0.05
**4**	121 ± 7	100 ± 8	129 ± 8	0.33 ± 0.03	0.29 ± 0.04	0.32 ± 0.05
**7**	127 ± 3	140 ± 30	130 ± 10	0.36 ± 0.01	0.23 ± 0.03	0.33 ± 0.06
**8**	126 ± 3	105 ± 5	140 ± 15	0.23 ± 0.02	0.31 ± 0.03	0.30 ± 0.05
**9**	125 ± 3	106 ± 7	133 ± 8	0.24 ± 0.01	0.32 ± 0.02	0.30 ± 0.05
**10**	127 ± 6	113 ± 3	132 ± 9	0.24 ± 0.01	0.29 ± 0.01	0.31 ± 0.07
**11**	133 ± 4	120 ± 20	137 ± 5	0.26 ± 0.02	0.27 ± 0.05	0.28 ± 0.03
**14**	130 ± 3	121 ± 7	144 ± 12	0.254 ± 0.04	0.29 ± 0.04	0.30 ± 0.04
**15**	136 ± 8	124 ± 11	148 ± 8	0.26 ± 0.02	0.27 ± 0.04	0.31 ± 0.04

Table S6: ζ-potential over time of NGs at different amount of HA (2.5%, 3.3%, 5.0%) and stored at 37 °C. Data are the mean ± standard deviation of three independent experiments.

	**ζ-potential (mV)**		
**t (day)**	**2.5%**	**3.3%**	**5.0%**
**0**	21.6 ± 0.4	22 ± 2	16.8 ± 0.9
**1**	21.8 ± 0.3	20 ± 1	19.1 ± 0.9
**2**	22.2 ± 0.6	18 ± 4	18.4 ± 1.2
**3**	24.3 ± 0.6	22.8 ± 0.8	20.0 ± 0.7
**4**	24.5 ± 0.9	23.2 ± 2	14 ± 2
**7**	25.9 ± 0.7	25.6 ± 0.4	24 ± 2
**8**	25.8 ± 0.7	24.7 ± 0.3	24.9 ± 1.2
**9**	26.8 ± 0.8	25.7 ± 0.8	26.4 ± 0.4
**10**	26.4 ± 0.8	26.5 ± 0.9	26 ± 2
**11**	27.5 ± 0.2	26.3 ± 0.6	27 ± 2
**14**	28 ± 2	28.2 ± 1.2	29.4 ± 0.2
**15**	27 ± 2	30 ± 2	28.8 ± 0.8
